# Solutions for Increased Adoption of Patient Portal Shared Access: A Human-Centered Design Approach Using the Double Diamond Model

**DOI:** 10.1055/a-2710-4288

**Published:** 2025-12-03

**Authors:** Danny L. Scerpella, Liz Salmi, Isabel Hurwitz, Amanda Norris, Kennedy McDaniel, Sara Epstein, Jennifer L. Wolff, Catherine M. DesRoches

**Affiliations:** 1 Department of Health Policy and Management, Johns Hopkins Bloomberg School of Public Health, Baltimore, Maryland, United States; 2 OpenNotes, Beth Israel Deaconess Medical Center, HVMA Annex, Boston, Massachusetts, United States; 3 Department of Research Administration, Johns Hopkins School of Nursing, Baltimore, Maryland, United States; 4 Providence Institute for Human Caring, Torrance, California, United States

**Keywords:** shared access, human centered design, consumer health informatics, caregivers, patient portal

## Abstract

**Background:**

Achieving digital health equity and proper use of identity credentials is crucial as reliance on electronic modalities increases. Proxy access—now increasingly referred to as *shared access* —is a widely available functionality that offers identity credentials to care partners who assist loved ones in navigating the electronic care delivery demands of patients with complex care needs. However, adoption of these tools has been hindered by complicated user interfaces and low awareness.

**Objective:**

Drawing on frameworks and principles rooted in human-centered design (HCD), we conducted an evaluation of a multisite quality improvement study designed to increase the awareness and adoption of shared access to patient portals for older adults and their care partners. Through feedback gathered from key informants, we identified barriers to the adoption of materials created for the parent quality improvement project, and synthesize additional implementation strategies from informant feedback to improve shared access.

**Methods:**

We employed the Double Diamond Model (DDM) of HCD to guide our research. The DDM includes engaging a diverse group of community partners—older adults, care partners, health care system leaders, communications professionals—through focus groups and individual interviews. Our process involved identifying pain points related to registration for shared access, then synthesizing these insights through inductive coding and affinity mapping to generate solutions.

**Results:**

An analysis of our community partner feedback revealed several themes, including the necessity for simplified patient portal registration, standardized terminology about shared access, and clear messaging strategies. A step-by-step video tutorial was developed as a prototype. The prototype was then implemented at a partner health system and received positive feedback, suggesting its potential for broader use.

**Discussion:**

These findings emphasize the importance of involving “end users” (patients, care partners, health care system leaders, communications professionals) in the evaluation and implementation of digital health tools. Approaching challenges with an HCD mindset helped our team identify barriers to shared access adoption and led to the development of a tangible resource (prototype and video). This project highlights the potential for HCD to drive improvements in digital health equity.

**Conclusion:**

This research demonstrates a practical application of HCD methods in developing effective solutions for enhancing shared access for older adults, and all people using patient portals.

## Background and Significance

Patient portals help patients view their health information and navigate electronic care delivery demands. 1 Patient portals have become a mainstream in care delivery interactions. 2 3 Yet millions of people with complex health needs are unable to independently manage their care. 4 5 Care partners—family members, friends, and community members who assist patients with daily activities—often find it difficult to access critical information about a patient's health and treatment. 6 Many health systems offer “shared access,” a functionality that allows patients to formally register a care partner to access their portal account using unique login credentials (username and password), 7 thus respecting patient preferences and need to involve other individuals in their care. 6 Shared access offers a range of benefits for care coordination and communication between care partners and clinicians; 8 however, adoption of this functionality has been limited and implementing effective processes at scale requires consideration of state laws, electronic health record (EHR) systems, and clinician opinions. 9 10 Portal user interfaces and registration processes are complicated and cumbersome, 8 and clinicians and staff cite complex registration processes, and infrequent use of shared access features such as tailoring care partner access permissions, scheduling, and messaging. 8 11 12 Additional complications include the lack of knowledge about registration processes by clinical staff, patient lack of clarity about the permissions and confidentiality granted with shared access, and concerns about equitable uptake of shared access for patients without traditional access to resources (also known as the “digital divide”). 6 12 13 14 Despite a significant growth of patient portal usage since 2020, 15 racial disparities persist where Black and Hispanic patients are 5% less likely to be offered access to the portal than White patients, while being 8% less likely to use the technology. 16 17 Overall, adoption of shared access is limited and gaps in implementation strategies merit attention.

Evidence-based guidelines and methodologies may support health care organizations in overcoming challenges in shared access uptake, however, implementing theoretical best practices in routine care is difficult. 18 There is an increasing call for research designs that engage multiple approaches to increase implementation efficacy including designs, which acknowledge the dynamic and relational nature of research and practice. 19 20 21 This is aligned with contemporary views on research priorities to support patient portal use and registration, specifically for closing gaps in adoption for diverse populations including among care partners. 22 One method is to develop and test implementation strategies in the environments that are intended to adopt them using human-centered design (HCD). 23 As defined by the International Organization for Standardization, HCD is a method that “aims to make systems usable and useful by focusing on the users, their needs and requirements…by applying human factors/ergonomics, usability knowledge, and techniques.” 24 HCD methods encourage participatory approaches that respect unique perspectives from individuals whose experiences have been affected by a given issue. 12 24 25 26 HCD processes result in “products” that are meaningful to end-users 27 and are developed by using cycles of rapid prototyping and iterative refinement. 26 By engaging clinicians, patients, and care partners, HCD may highlight useful insights for improving shared access derived from end-user feedback.

We sought to explore care partner perspectives on engagement with patient portals, in conjunction with findings from a multisite demonstration of a quality improvement project, and explored additional solutions that could increase shared access uptake. The parent quality improvement project drew on insights from an investigation into systemic challenges of shared access, 8 28 and a survey of participants attempting to sign up for shared access. 29 Additionally, a set of patient portal educational materials (brochures, posters, website, and implementation toolkits) was co-designed with patients, care partners, clinicians, and staff from three partner health care organizations. 12

## Objectives

The objectives of the HCD process and prototypes described in this manuscript are intended to build upon the parent study by:

1. Collecting feedback on shared access processes and educational materials developed in our previous project from key informants outside of our partner health organizations and
2. Describing a design methodology for evaluating key informant feedback to recommend organizational strategies to increase public awareness and use of shared portal access functionality.

## Methods

We used the Double Diamond Model (DDM) process of HCD as a framework to guide our approach to eliciting feedback from key informants. Originally developed by the British Design Council in 2004, 30 the DDM is a standardized design process that places a priority on contextual factors to identify core issues and find solutions. The DDM was selected for this project for its emphasis on innovative thinking and on flexible methodological processes allowing rapid response to and iteration from evolving feedback. 31 The DDM was prioritized because it is a simple process that allows respondents to effectively participate in the design process regardless of educational or theoretical background, 32 and it integrates the lived experience perspective in the co-evaluation and co-creation of solutions that are meaningful to end users. The DDM consists of four phases: *Discover* , *Define* , *Develop* , and *Deliver* . The *Discover* phase prioritizes collecting community insights and ideas through interviews with an emphasis on “divergent thinking,” or outside-the-box thinking, for solutions and innovation. 31 33 The *Define* phase involves homing in on the definition of a problem based on feedback from community members, as well as systematically synthesizing information. Inductive coding 34 35 is used to summarize feedback and thematically link these with research objectives to develop a model or theory based on experiences and processes within the text and observations from respondents. The *Develop* stage is used to formulate potential solutions for discussion and exploration through repeated rounds of iteration. Finally, the *Deliver* phase refers to selecting a single solution and evaluating it while removing and improving individual components that do not function as intended. The DDM focuses on stages of both divergent ( *Discover, Develop* ) and convergent thinking ( *Define, Deliver* ) which are used to prioritize the greatest spread of ideas, and then collating these ideas into a unified, comprehensive solution. 31

### Interviews

Key informant research is the involvement of patients and other community partners in the research process to highlight the importance of “incorporating the knowledge and experiences of those closest to the issue being researched.” 36 37 In our process, we recruited key informants whose background, roles, and experiences mirrored the perspectives captured in the parent quality improvement project, but included individuals broadly recruited from outside of the three partner health organizations. Invitations for focus groups were sent to potential key informants, and individuals who were unable to attend a focus group we offered an option to participate in 1-on-1 conversations. Informants included:

- **Patients and/or care partners** who expressed interest in shared access *for themselves* (as patients) or *for another person* (e.g., family member).
- **Project implementers** (including physicians) who had decisional power in their roles to lead new health IT-related initiatives. These individuals held titles like chief medical information officer or were involved in patient portal decisions and had experience with leading change management initiatives.
- **Connectors/influencers** (including physicians) who were broad, influential advocates with strong connections to health systems. These individuals were health care professionals and/or executives knowledgeable about the history, policy and research around patient access to information (e.g., HIPAA, open notes, 21 ^st^ Century Cures Act, information blocking rule).
- **Marketing and communications professionals** who worked inside of health systems or were communications professionals for health IT professional societies. They often created materials and led communications campaigns about adopting shared access messaging.

Patient and care partner participants were identified through the existing Coalition for Care Partners network—a joint collaboration between Johns Hopkins School of Public Health and OpenNotes at Beth Israel Deaconess Medical Center—and social media channels run by the OpenNotes communications and dissemination team. 38 39 OpenNotes has had more than a decade of experience in research implementation and dissemination and maintains a strong network of patient, care partner, and clinician collaborators. 40 As a result, OpenNotes was able to identify implementers and connectors through existing relationships and worked with Johns Hopkins colleagues to obtain referrals to additional implementers and connectors. Similarly, marketing/communications staff were identified through snowball sampling within the OpenNotes and Johns Hopkins communications staff and referrals from colleagues.

The interview guides were designed to assess the most critical pain points around shared access for each group of key informants and illicit creative solutions to those challenges. Additional questions were designed to evaluate the educational materials developed as part of the parent quality improvement project. 12 Focus group/interview questions included:

- Is shared access important to you? Why or why not?
- Have you faced any challenges in learning about or setting up proxy access?
- What might prevent people from setting up proxy access?
- Beyond the materials reviewed today, are there other ways we can promote proxy access?

While some questions were consistent across groups, interview guides were tailored for each type of key informant. Interviews drew on knowledge of the individual's background and/or role and prioritized personal experiences regarding the topic of shared access to the patient portal.

All participants were sent information about the interviews and their role in the research in a prospectus agreement shared 48 hours ahead of a focus group or interview. This information was also reviewed at the start of each interview session. Transcripts of the conversations were recorded, with consent, for analytic purposes only. In addition, at least one team member was available to take minutes at each session. Patient and care partner participants were offered $100 in honoraria. This work was reviewed and approved by the Beth Israel Deaconess Medical Center Committee on Clinical Investigations and was assigned protocol no.: 2022P000020.

### Mural

To plan, prototype, organize, and synthesize the large number of insights developed during focus group and individual interview sessions with end users, the study team utilized Mural. 41 Mural is a virtual and visual workspace designed to facilitate efficient teamwork allowing for collaborative participation and facilitation using outlining, diagramming, and workflow tools. Logical relationships between observations and themes were visualized in Mural using a variety of approaches such as idea matrices and affinity mapping. Affinity mapping is a design approach used to visually interpret data to reveal connections and themes through collaborative discussion. 42 This method favors collaboration over structure that allows users greater flexibility to synthesize multiple data sources into agreed upon categorizations.

## Results

Feedback from key informants was synthesized according to prevalent themes with the goal of identifying potential solutions, which were rank-prioritized according to the highest probability of effect and success.

### Discover

In total, 47 key informants were recruited to provide feedback ( Table 1 ). Of the informants, 20 participated in one-on-one interviews (each 30 minutes in length), and five focus groups were conducted with four to six participants (each 90 minutes in length).

**Table 1:** Focus groups and 1-on-1 interview participation

Partner roles (personas)	No. in focus group	No. 1-on-1 interviews	Total participants
Project implementers	5	7	12
Health care marketing and communications staff	4	2	6
Patients	6	3	10
Care partners	5	6	11
Connectors and influencers	6	2	8

**Project Implementers** , which among them included nine physicians, emphasized why shared access was important and highlighted systemic strategies for increasing uptake, such as mandatory sign-up; dedicated resources for patients who want shared access; removing logistical barriers and increasing ease of granting shared access; standardization of terminology (proxy vs. shared access); and creating a spectrum of solutions relevant to health systems at varying levels of willingness/ability to enact a change.

**Marketing and Communication** professionals emphasized the importance of standardized terminology (e.g., “shared access” vs. “proxy access”) to help with their efforts. Additionally, they desired clearer messaging to patients about the value of shared access; easy-to-understand and follow instructions with pictures; wanted to see care partners prioritized in messaging about the importance of shared access; and stated that educating clinic staff about shared access would make the concept more relevant to their own lives and increase personal buy-in and investment.

**Patients** endorsed guided facilitation of enrollment in shared access, such as staff-guided step-by-step instructions; reminders for patients at multiple contact points (e.g., in the waiting room, after visits, through portal messages); and automated reminders within the patient portal. Patients recognized inconsistencies of shared access messaging across health systems and agreed that varied terms should be standardized (i.e., “shared access” instead of “proxy access”).

**Care Partners** emphasized frustration with interoperability and changing requirements for shared access between health care systems and expressed the need for a more convincing reason *to* *not use* patient login credentials when accessing a loved one's portal information. Care partners suggested a preference for account management tools similar to existing applications, such as Netflix (i.e., one main account with sub-account designations), with granular controls for patients to decide who has specific access permissions to their records.

**Connectors,** which included three physicians, named additional interest groups and stakeholders who could advance shared access through advocacy, policy, marketing, data analysis, and economics; endorsed monetary incentives for health organizations to increase shared access; and noted innovative technology companies may provide inspiration in the design of a universal system of shared access based on modern accessibility standards.

### Define

After completing focus groups and 1-on-1 interviews, we used affinity mapping and inductive coding to organize feedback into thematic groupings to identify patterns of feedback across all the information collected. The study team used the results from these processes to define themes and contextualize the most prevalent viewpoints from informants ( Table 2 ). These themes indicated that solutions for increasing the utilization of shared access need to: (1) include acknowledgement of clinician and staff overload; (2) rely on simple and effective processes and workflows that clearly demonstrate value and ease of use; (3) utilize technology and existing functions within the EHR system and connected organizational resources; and (4) function as a natural extension of clinician–patient relationships that invoke emotional understanding and safety in users through storytelling and “warm handoffs.”

**Table 2:** Human-centered design focus group themes

Acknowledging staff burnout	Reducing complexity	Rethinking shared access intervention adoption process	Facilitating shared access adoption beyond materials
• Sustainable intervention for overworked staff • Supply necessary support and resources	• Create a workflow that is easier than password sharing • Demonstrate the benefits of separate log-in information for patient the care partner • Expand privacy settings with clear explanations	• Successful adoption of intervention without a research team • Inclusion of technology for buy-in • Involve EHR systems and legislation	• Warm handoff (via an instructional video or staff member) • Inclusion of storytelling and emotional drivers

Abbreviation: EHR, electronic health record.

### Develop

**Fig. 1:**
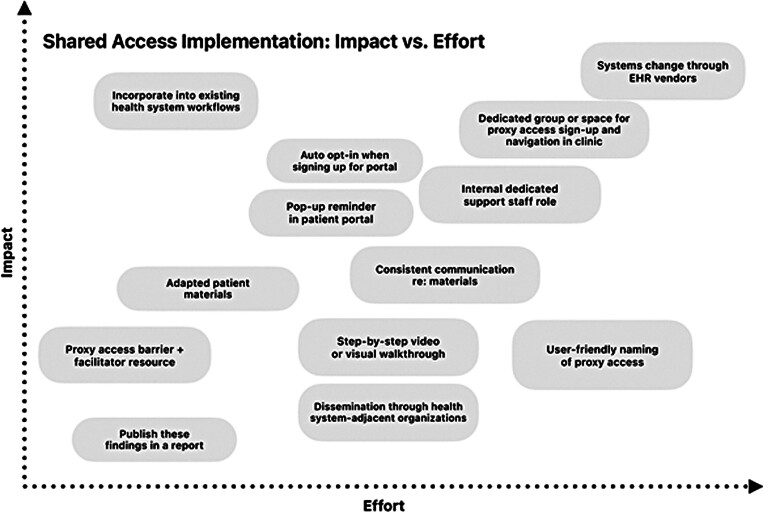
Shared access prioritization idea matrix.

The study team collaborated regularly via Mural to collaborate and draft potential solutions responding to the themes synthesized during the *Define* phase. Proposed solutions were modified, combined, or eliminated through iterative discussion and consensus among team members until the remaining options represented ideas that were sufficiently distinct and responsive to key themes. An idea matrix was developed to organize a full set of 13 potential solutions according to the magnitude of the potential effect on shared access uptake for patients and utility for the health system, and the amount of effort each solution would require to implement ( Fig. 1 ). The HCD study team presented the idea matrix in an all-site meeting consisting of leaders from across three-partner health care organizations involved in the parent quality improvement study. 12 Attendees were asked to provide feedback on the feasibility of the proposed solutions and the ease of implementation within their existing health system context. The HCD team presented each idea on the matrix starting with the most effective solution, which would take the most effort, down to the least effective solution, which would require the least effort. Each idea was presented, and the HCD team detailed their rationale for placement on the matrix. The attendees were asked to discuss each idea with specific consideration to the feasibility of the idea, its potential to change shared access behavior, the ease of implementation within their existing health system context, and the cost to implement. The solution with the greatest effect that would require a high amount of effort was wholesale system changes enacted through EHR vendors (derived from “Rethinking Shared Access Intervention Adoption Process” in Table 2 ), which the group recognized as not being feasible within the scope or budget of this project. After presenting all possible ideas to leadership, a step-by-step video on how to grant shared access through the MyChart patient portal was selected (derived from “Facilitating Shared Access Adoption Beyond Materials” in Table 2 ) due to the ease of prototyping using consumer grade technology and software (feasibility), available resources at partner sites (potential to change), and similar shared access videos which could be modified and used to guide progress (cost to implement).

### Deliver

**Fig. 2:**
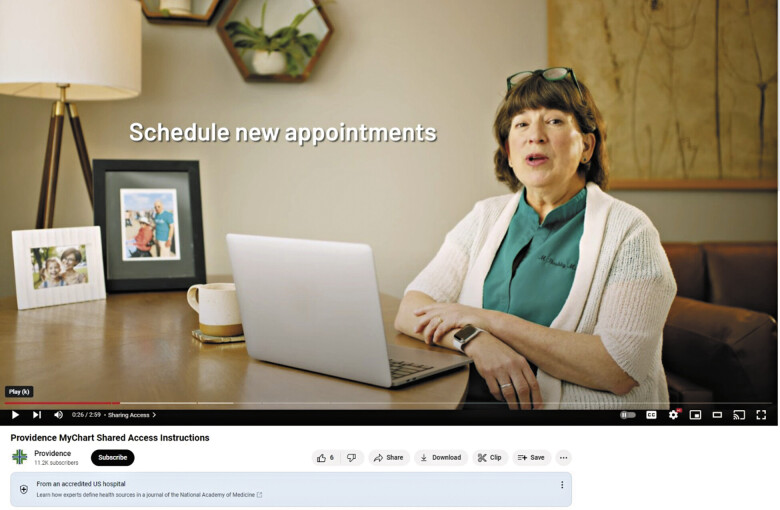
Screenshot of the official shared access instructional video on the Providence YouTube page.

The study team developed a low fidelity prototype of the shared access video. Prototype activities included a draft script and rough storyboard for an instructional video. We used an existing shared access instructional video created by Epic 43 as a reference; however, we modified the reference to place a priority on patient perspectives, health system-specific EHR functionality, customization, and branding. Using a preexisting research relationship developed as a demonstration site during the parent study, the study team initiated a collaboration with Providence health system. 12 Leadership at Providence Institute for Human Caring supported the implementation of the instructional video with live navigation of their portal narrated by a Providence clinician ( Fig. 2 ).

A video script was developed to contextualize instructions from a clinician perspective to replicate a trusted patient-provider relationship. In partnership with the study team, leadership at Providence selected a clinician—who also had experience using shared access as a care partner. The study team included the clinician in the design and review process for the script to increase accuracy, simplify language for patients, and add personal insights to develop storytelling elements. Providence asked their internal marketing and communications experts—some of whom had participated in the focus groups described earlier—to co-design the implementation with the study team. Storyboards for the video were developed and shared for review, and the study team provided feedback to the marketing team.

The study team revised the script to align with the final storyboard. Providence engaged an in-house staff for production, including a professional videographer and makeup artist. The filming of framing and education segments with the clinician took place in October 2023. Click-by-click navigation on the health system portal was captured for shared access sign-up to visually model the exact steps patients would need to take to complete the process on the Providence portal. A voiceover by the physician featured in the video was later added to these segments to explain each action in full detail. A draft video underwent three rounds of review, with editing supervised by health system leadership and the study team, before the video was finalized. Edits mainly included notes for pacing of the video and the length of time portal instructions were shown on screen. The video was released to the Providence YouTube channel to coincide with National Caregivers' Month in November and can be found at the cited link. 44

## Discussion

This study offers a unique contribution to the field of clinical informatics by applying HCD to the specific challenge of shared access to patient portals—an area that has received limited attention in both HCD and implementation science literature. 12 45 Unlike traditional “proxy access” models, our approach reframes the concept as “shared access,” emphasizing relational dynamics and patient empowerment. Existing dissemination and implementation strategies prioritize engagement with end users, dissemination agents, and interpersonal communication. 45 HCD is not well-integrated into the mainstream development of implementation science; however, it is an area of study which is growing in importance. 19 This work collected perspectives about shared access from a diverse group of key informants and evaluated a prior quality improvement project. 9 The authors used the DDM to understand contextual factors, help identify core issues, and develop solutions for improving receptivity to shared access. While our focus group participants shared favorable views of shared access materials from the parent study, additional ideas were suggested for further exploration. Several solutions were derived from the HCD process, and we were successful in partnering with a large not for profit U.S. health system to rapidly prototype and implement a guided, simplified instructional video for patients seeking to learn about and obtain shared access to their patient health records.

This project is a novel application of HCD, which has not been richly applied to the challenge of shared access to the patient portal and is not often applied in implementation science in general. This approach allowed us to create prototype ideas that were attractive to real world care delivery organizations and addressed feedback from key informant perspectives at the organizational and patient/care partner level. The video prototype addressed the 4 key themes derived from informant interviews by: (1) acknowledging clinic personnel overload and lack of dedicated time (Acknowledging Staff Burnout); (2) presenting helpful information in a clear and understandable manner (Reducing Complexity); (3) utilizing existing technology that is easily accessed (Rethinking Shared Access Intervention Adoption Process); and (4) connecting with patients by using a practicing clinician within Providence health system in the video (Facilitating Shared Access Adoption Beyond Materials). After the video was published online, additional health systems expressed interest in reproducing its content, which suggests potential to further disseminate the shared access video outside of the parent study project.

This work validates previous research conducted by the parent quality improvement study 12 who also derived insights from informants for topics such as cumbersome technical processes for obtaining shared access, the need for clear instructions, and considerations for overworked clinical staff. Outcomes from the parent study also showed that many patients were unaware of shared access and that the quality improvement study did not significantly increase new shared access registration. 28 The video prototype responds to a need for additional solutions to facilitate and encourage shared access and is intended to be repurposed by other health systems through simple adaptation of branding, color palettes, and most importantly, functionality specific to each EHR. The video prototype expands future directions outlined in previous work and provides a targeted and specific product that educates and instructs patients and their care partners on the exact process for signing up for shared access.

Two major lessons were learned from this project. First, health systems support efforts to engage in relatively low-effort initiatives that have potential for great effect and seem to show willingness to collaborate to incorporate key informant perspectives. Second, feedback from informant interviews can be leveraged to improve messaging to patients and care partners. For instance, participants from informant groups indicated the need for instructional resources, easy-to-use privacy controls, and personal, guided facilitation as well as a movement away from confusing terms such as “proxy access.” The video prototype includes visual, step-by-step instructions delivered by a relatable clinician/care partner, combined with a simplified script that avoids confusing terminology and was co-written by the health system, an experienced clinician, and a HCD research team.

There are several limitations to this research. We initially aimed to recruit eight participants per focus group, targeting a total of 40 participants. However, coordinating group sessions with diverse stakeholders proved challenging ( Table 1 ). To address potential gaps in representation, we conducted an additional 20 one-on-one interviews to capture a broader range of perspectives. Ultimately, we included insights from 46 individuals. While this approach allowed us to meet our overall recruitment goal, we acknowledge that the shift from group discussions to individual interviews may have influenced the nature of the information shared with the HCD team. Additionally, while participants were recruited through institutional networks and social media, we recognize the potential for selection bias toward individuals who are already familiar with or interested in patient portals, which may limit generalizability. While this project successfully evaluated the educational materials from the parent quality improvement study through informant interviews, it evolved beyond evaluation into exploring additional solutions for shared access beyond what was originally developed. Future efforts to design digital health interventions may find value in HCD methods that encourage evolution of ideas and rapid cycle prototyping. Ultimately, systematic change is necessary to address complex sign-up procedures that require extensive forms to be completed in-person or confusing multistep authorization procedures that may be burdensome for some patients. The video prototype attempts to respond to these issues by being tailored to the health system for which it is implemented. Clinics adopting a similar video need step-by-step instructions specific to their own shared access processes, and all organizations adapting this approach would need to periodically revisit the video to ensure accuracy to current policies and EHR functionality. An instructional video may not benefit all patients including those who are not English-speaking, and it is unclear at present the full effect of the video prototype. This study was not designed to evaluate whether proposed solutions affect care partner engagement with shared access functionality. As such, additional work should be done to target implementation of these videos to track adoption, engagement, sustainability, and a strategy for visibility to patients across the system. Future research should prioritize multimodal interventions for shared access including print materials, instructional videos, interoperable EHR standards and practices, and advocacy to state-level agencies and health systems to emphasize the barriers to shared access.

## Conclusion

We successfully evaluated a multisite quality improvement study by utilizing an HCD methodology intended to evaluate key informant feedback on organizational strategies to increase public awareness and use of shared portal access functionality. This research demonstrates a practical process for eliciting, codifying, and rapidly developing efficient and effective responses to key informants on the topic of shared access using HCD methods. We demonstrate the feasibility and importance of working with clinicians, patients, care partners, and health systems to utilize HCD methodologies that help develop thematic analysis and identify solutions that can be adopted with minimal barriers. By co-designing a low-burden, scalable instructional video with input from patients, care partners, and health system stakeholders, we demonstrate how HCD can be used not only to identify barriers but also to rapidly prototype and implement solutions that are adaptable across diverse care settings. This work bridges a critical gap between user-centered design and real-world implementation, offering a replicable model for future digital health interventions.

## Clinical Relevance Statement

This study demonstrates how HCD methods can be used to identify novel solutions for simplifying shared access to patient portals and benefit patients and care partners. The HCD process resulted in a prototype video developed in collaboration with a health system to address barriers to adoption that were revealed through informant interviews such as complex registration processes and low adoption rates. HCD methodologies can help health systems identify practical solutions for improving care coordination through patient portals.

## Multiple-Choice Questions

1. What was the primary goal of applying Human-Centered Design (HCD) in this study?
  - Increase health system revenue
  - Improve the design of patient portal interfaces
  - Utilize HCD methods to synthesize, understand, identify, and develop solutions for shared access
  - Enhance patient access to test results
  **Correct Answer:** The correct answer is option c. This study aimed to describe a methodology for evaluating key informant feedback about shared portal access by applying HCD methodologies such as the Double Diamond Model.
2. Which group was not included in the key informants interviewed in this study?
  - Patients and care partners
  - Health care marketing and communications professionals
  - Health system executives
  - The payors of health services (e.g., insurers, CMS)
  **Correct Answer:** The correct answer is option d. Payors were not included. This study aimed to evaluate feedback from end users of shared portal access functionality including patients, organizational leadership, and marketing.
3. What is one of the major challenges the study identified in adopting shared access to patient portals?
  - Lack of clinician interest
  - Complexity of portal interfaces and registration processes
  - High costs associated with shared access technology
  - Insufficient EHR functionality
  **Correct Answer:** The correct answer is option b. Complexity of portal interfaces and registration processes were most relevant to key informants in systems where shared portal access functionality exists but is not widely adopted.
4. How did the researchers address the challenge of complex registration processes for shared access?
  - By simplifying the portal software
  - By developing an instructional video prototype
  - By conducting additional user interface training for clinicians
  - By reducing the number of required forms
  **Correct Answer:** The correct answer is option b. The development of an instructional video prototype was selected by health organizational leadership due to ease of prototyping using consumer grade technology (feasibility), available resources at partner sites (potential to change), and existence of similar products which could be adapted (cost to implement).
5. What human-centered design methodology did the study use to guide the development process?
  - Lean Startup
  - Six Sigma
  - Double Diamond Model
  - Agile Development
  **Correct Answer:** The correct answer is option c. The Double Diamond Model was selected for its emphasis on flexible methodological processes for rapid iteration. This model allowed for meaningful participation of key informants in the design process without the need for specialized education or training.
